# Supplementary figures and images for: LAMP-coupled CRISPR-Cas12a assays: A promising new tool for molecular diagnosis of leishmaniasis
Source: PLoS Negl Trop Dis. 2026 Feb 13;20(2):e0013456. doi: 10.1371/journal.pntd.0013456 (PMC12923138; doi:10.1371/journal.pntd.0013456)

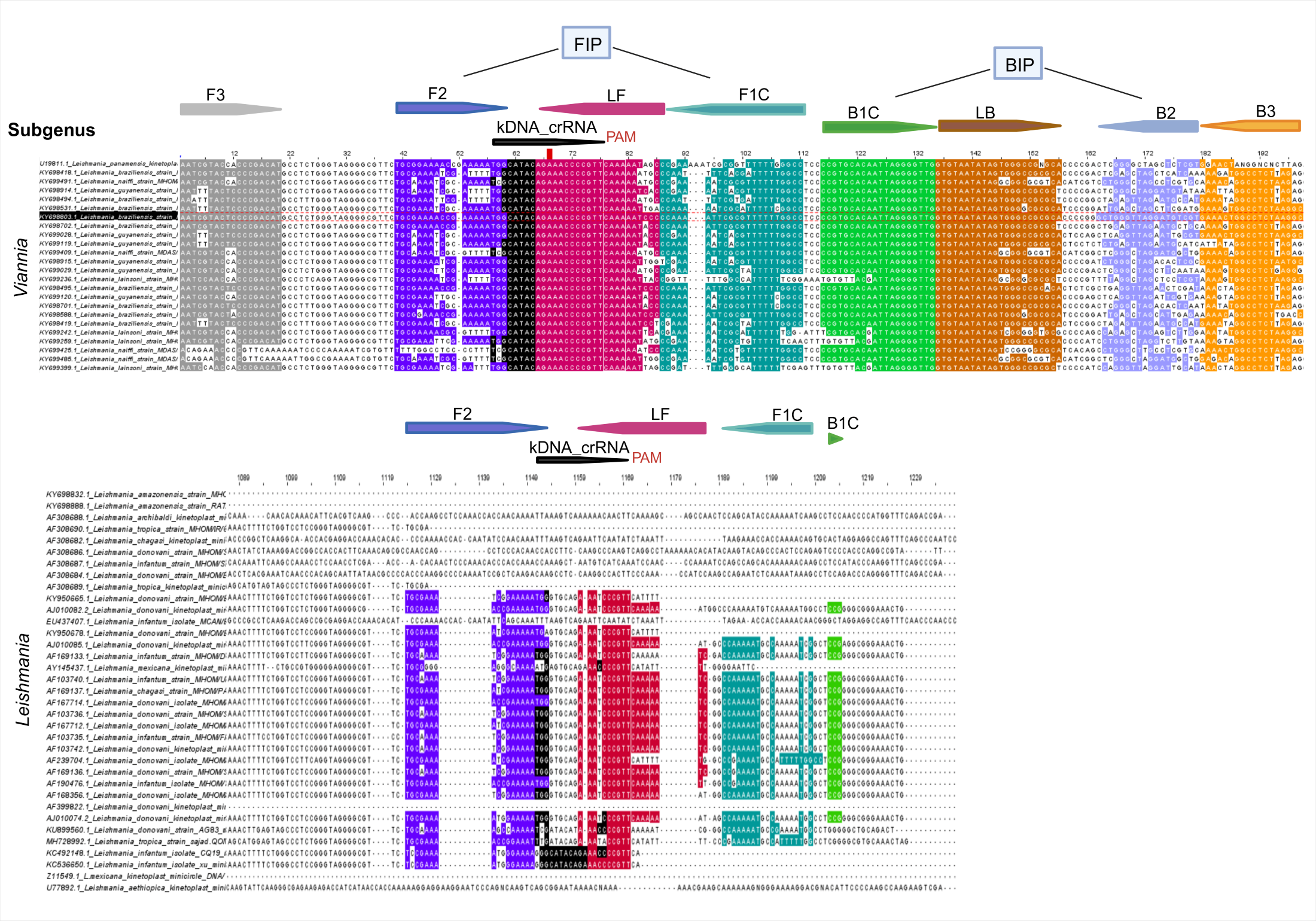

Supplement: S1 Fig — A total of 24 L. (Viannia) and 35 L. (Leishmania) sequences were aligned using Clustal Omega (alignments were performed separately for each subgenus) and visualized in Jalview v2.11.4.1. The locations of primer binding regions as well as of the crRNA target site and PAM sequence in the target DNA are highlighted with colors to indicate sequence conservation within the L. (Viannia) subgenus and sequence divergence within the L. (Leishmania) subgenus. Nucleotide variants are represented by spaces in the alignment. The kDNA minicircle sequence from the L. (V.) braziliensis M2904 strain (GenBank accession no. KY698803.1) exhibited 100% identity across all primer and crRNA target regions. The sequences shown are from selected representative strains/isolates of a given Leishmania species; additional species and sequences can be examined in the full alignments included in the S1 File. Figure created in BioRender. Upc, C. (2026) https://BioRender.com/0hntnbn, with permission to sublicense under CC-BY 4.0. (TIF) [file pntd.0013456.s001.tif]

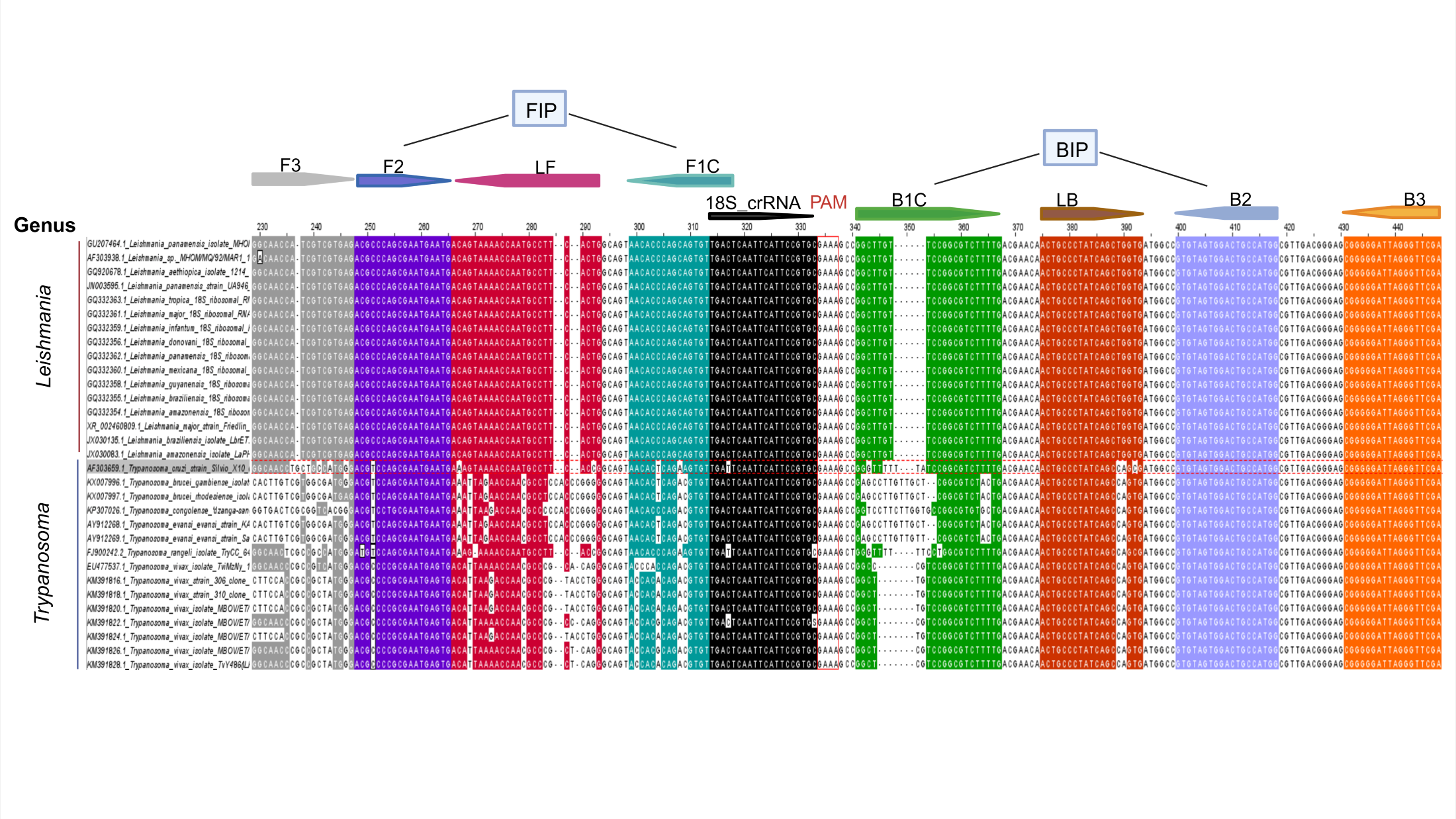

Supplement: S2 Fig — A total of 31 sequences (16 from Leishmania spp. and 15 from Trypanosoma spp.) were aligned using Clustal Omega and visualized in Jalview v2.11.4.1. Primer binding regions as well as the crRNA target site and PAM sequence in the target DNA are highlighted with colors to indicate sequence conservation within the Leishmania genus and sequence variations within the Trypanosoma genus. Nucleotide variants are represented by spaces in the alignment. The sequences shown are from selected representative strains/isolates of a given species; additional species and sequences can be examined in the full alignments included in the S2 File. Figure created in BioRender. Upc, C. (2026) https://BioRender.com/54b2q2n, with permission to sublicense under CC-BY 4.0. (TIF) [file pntd.0013456.s002.tif]

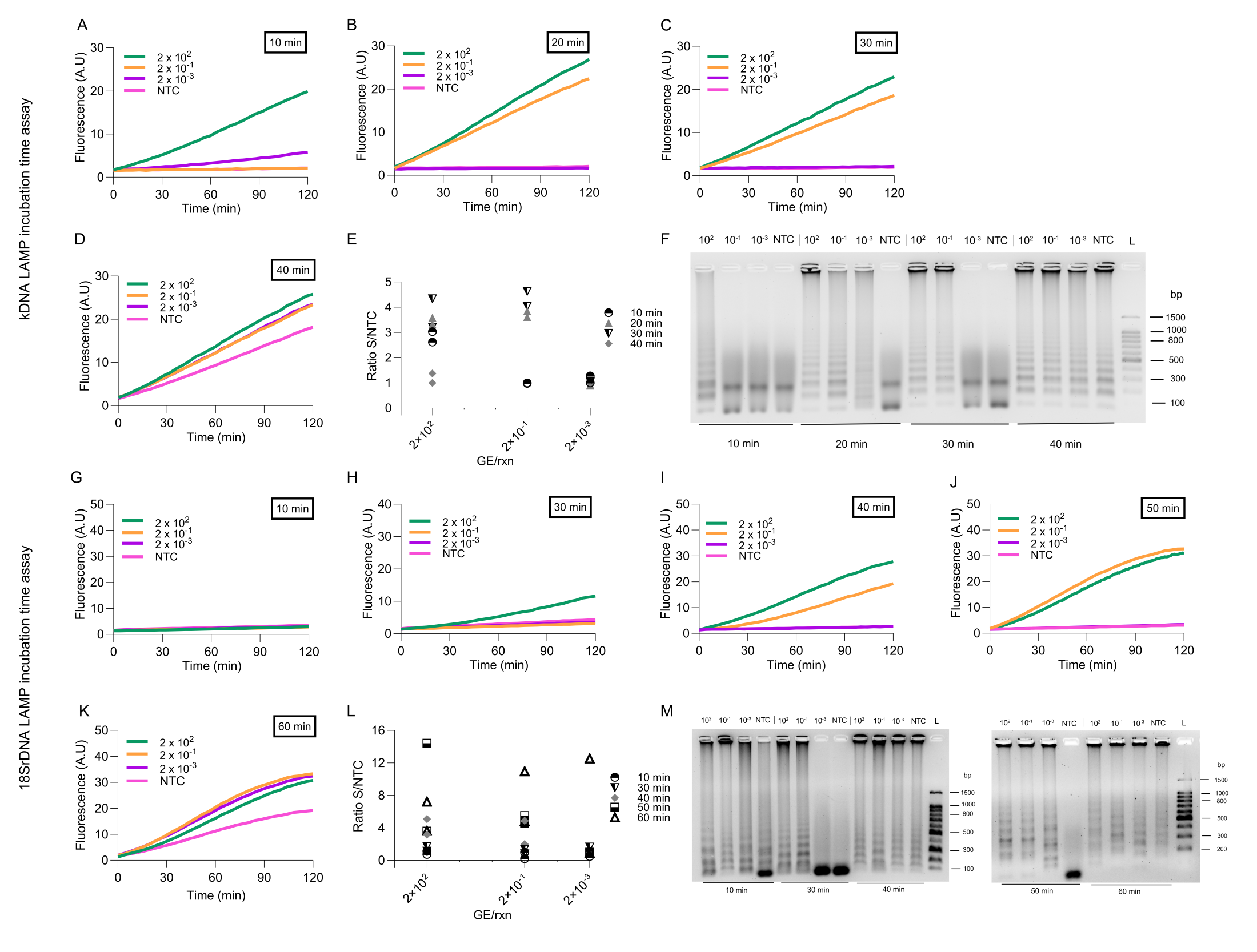

Supplement: S3 Fig — To determine the minimum necessary incubation time of the LAMP reaction for consistent DNA amplification, tests were performed at different reaction times for Leishmania kDNA (10, 20, 30, and 40 min) and 18S rDNA (10, 30, 40, 50, and 60 min) targets. The number and text enclosed in a rectangle indicates the LAMP reaction time. The amplification efficiency was assessed using varying amounts of template DNA (L. braziliensis M2904 gDNA), corresponding to approximately 2 × 10^2^, 2 × 10^-1^, and 2 × 10^-3^ genome equivalents. A negative control (NTC) reaction was tested in parallel. Raw fluorescence signals from Cas12a reactions are shown for kDNA (A-D) and 18S rDNA (G-K) for one representative technical replicate. Normalized data (fluorescence ratio at t = 25 min) from two technical replicates are shown in panels E (kDNA) and L (18S rDNA). A fluorescence ratio ≥ 2 is considered detected. Fluorescence measurements in this figure were taken on the Varioskan LUX plate reader. LAMP reaction products (5 µL each) of kDNA (F) and 18S rDNA (M) were analyzed by 2% agarose gel electrophoresis ran at 100 V for 1 h using SYBR Gold staining. L, 100 bp DNA ladder (ABclonal). (TIF) [file pntd.0013456.s003.tif]

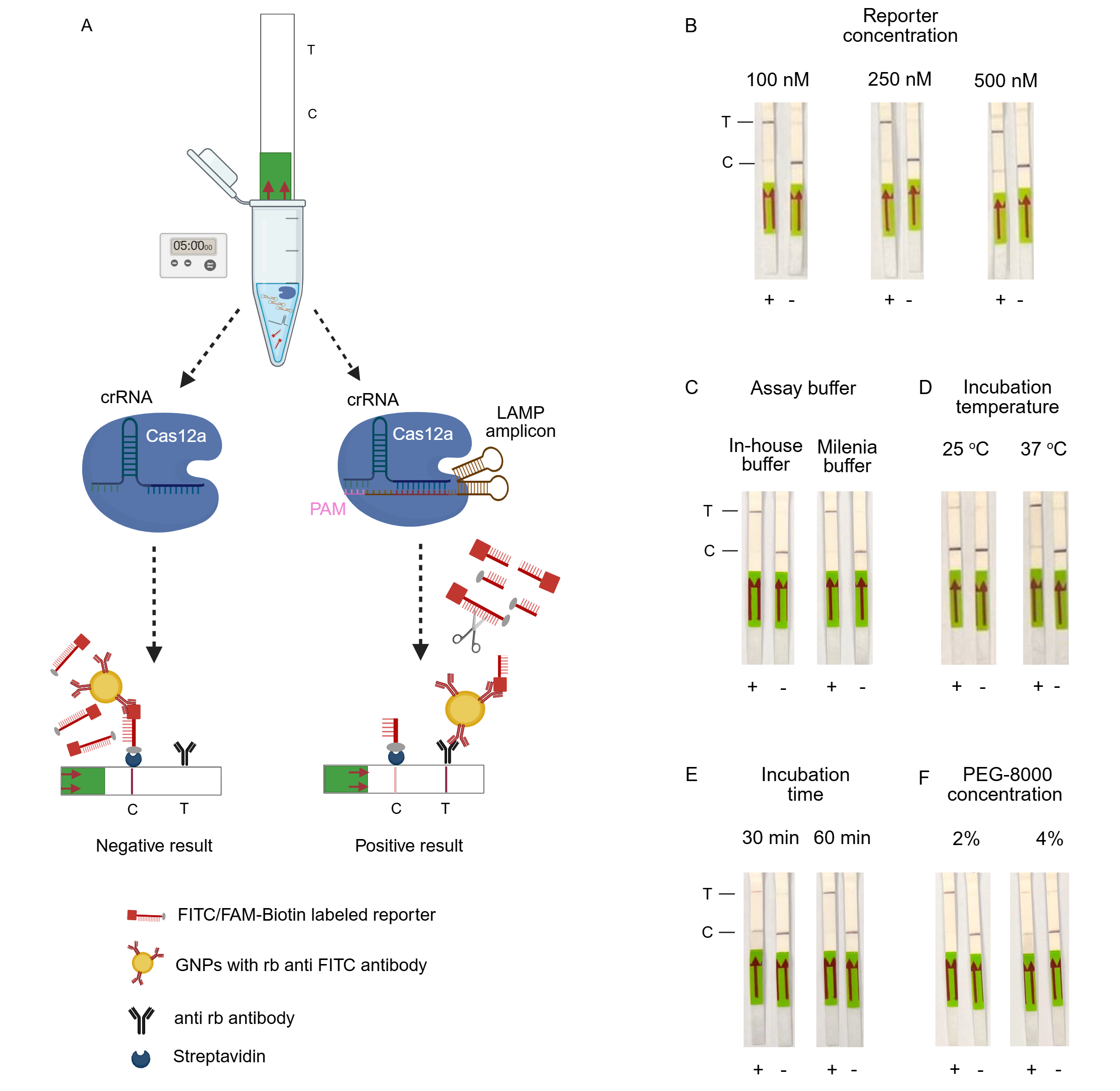

Supplement: S4 Fig — (A) Design of the Milenia GenLine HybriDetect test strips and readout interpretation. (Left) In the absence of the specific genetic target, the intact biotin-FAM labeled reporter molecules flow to the control line (C-line). This is interpreted as a negative test result. (Right) Upon recognition of the genetic target, the CRISPR RNP complex cleaves the reporter molecules, which flow to the test line (T-line). The signal intensity of the C-line is weakened. If cleavage of the reporter molecules is partial, both the C-line and T-line may appear with comparable intensity. Both scenarios are interpreted as a positive test result. Figure adapted from [113] and created in BioRender. Upc, C. (2026) https://BioRender.com/bju6fw7, with permission to sublicense under CC-BY 4.0. (B-F) Different parameters influencing the LFA readout performance were evaluated to achieve consistent results: reporter concentration (B), assay buffer (C), incubation temperature (D) and incubation time (E) of the Cas12a assay, and the concentration of PEG-8000 (F). One positive control (2 × 10^2^ GE from L. braziliensis M2904 gDNA) and a negative control (NTC) were tested. Results shown here correspond to the kDNA LAMP-CRISPR assay. Photos taken by the authors. (TIF) [file pntd.0013456.s004.tif]

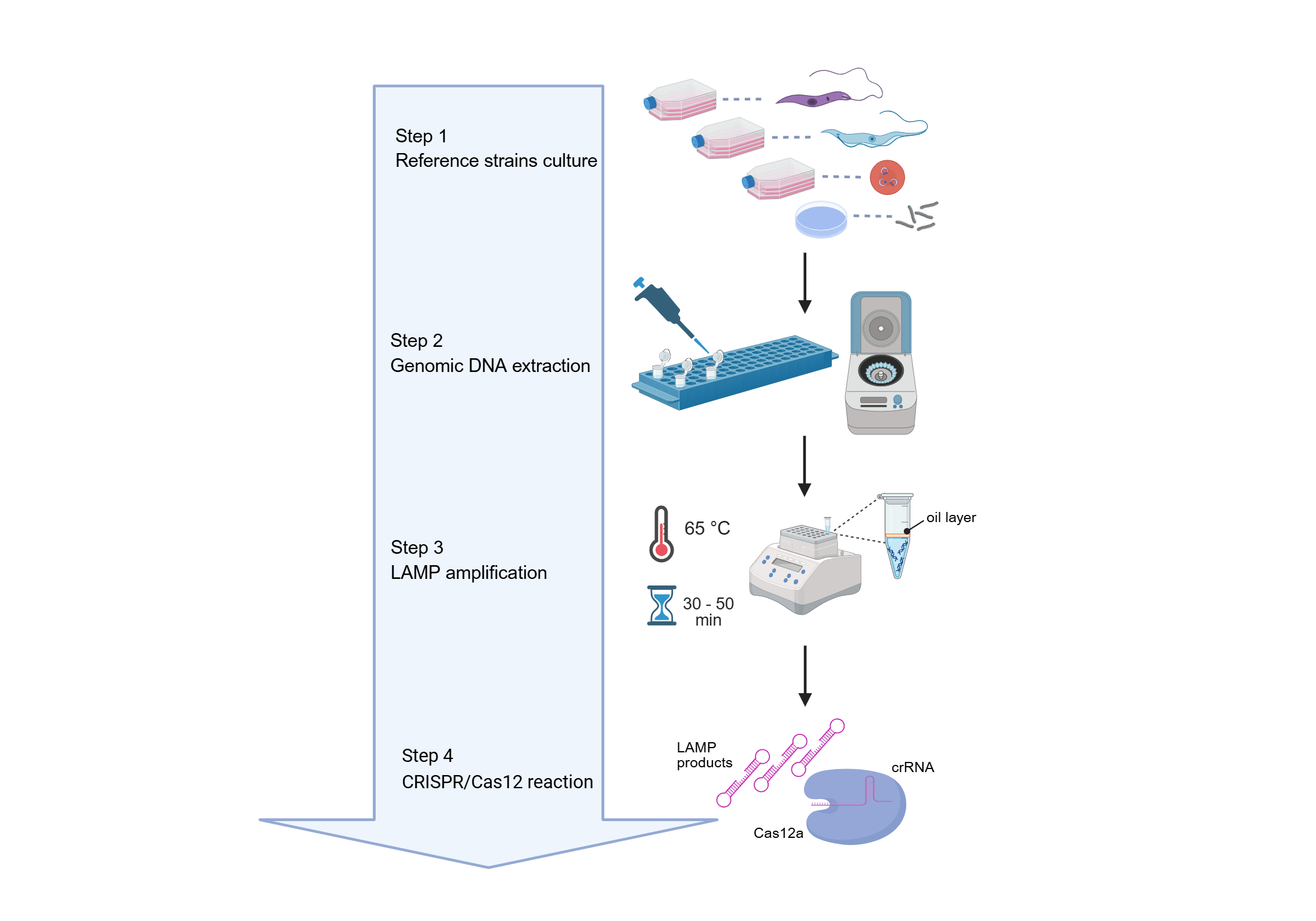

Supplement: S5 Fig — Figure created in BioRender. Upc, C. (2026) https://BioRender.com/f9m6gf2, with permission to sublicense under CC-BY 4.0. (TIF) [file pntd.0013456.s005.tif]

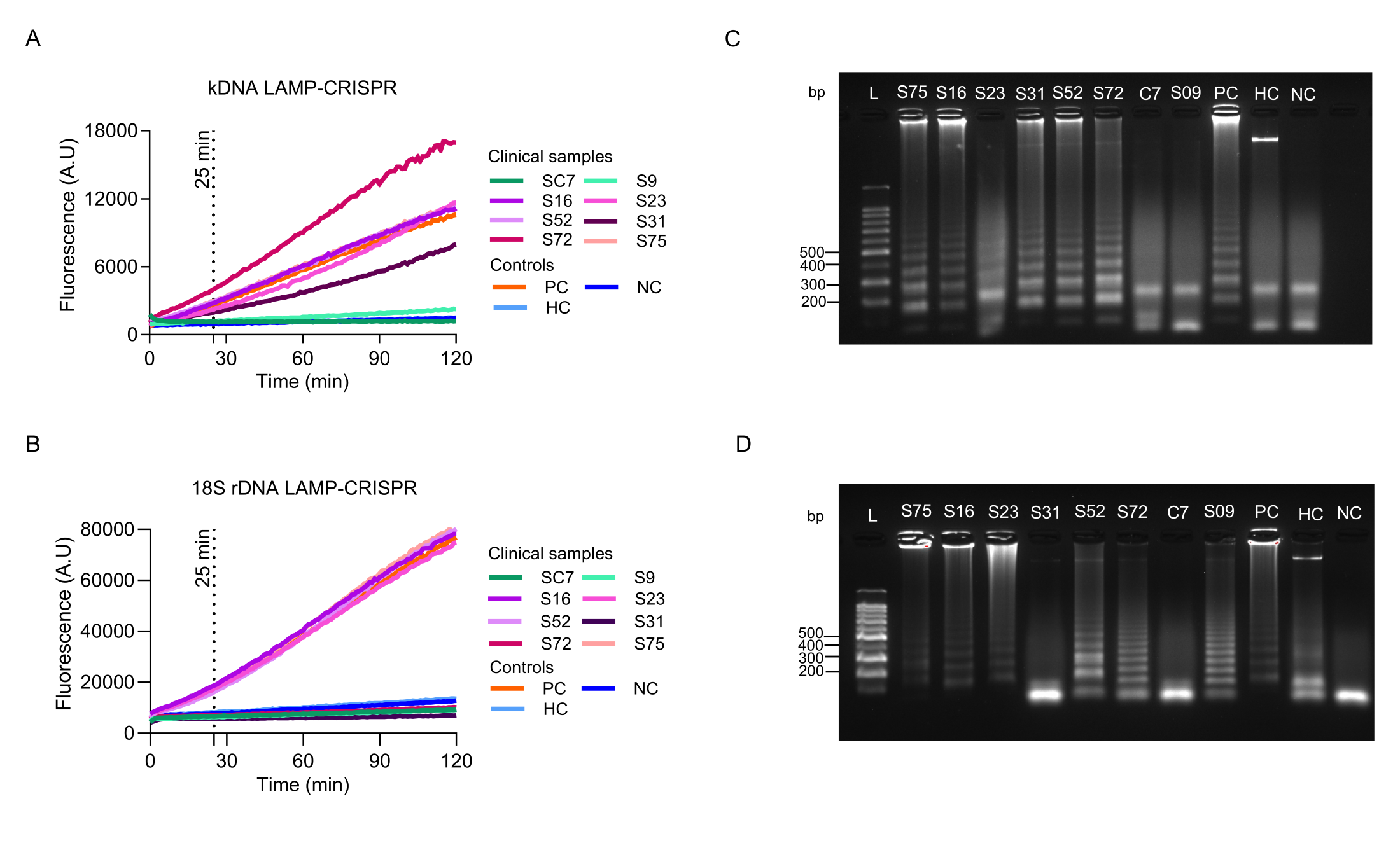

Supplement: S6 Fig — Raw fluorescence curves generated by Cas12a detection of LAMP amplicons of Leishmania kDNA (A) and 18S rDNA (B) in a representative group of clinical samples harboring varying parasite load levels (see S1 Data) and ran in the same assay plate per genomic target. Out of the 8 tested samples shown, 6 showed robust fluorescence curves indicating the presence of Leishmania kDNA molecules (panel A; samples coded S16, S23, S31, S52, S72, and S75) whereas the 18S rDNA target was detected in 4 of them (panel B; samples coded S16, S23, S52, and S75). The respective LAMP amplicons (5 µL) of kDNA (C) or 18S rDNA (D) were analyzed by 2% agarose gel electrophoresis (100 V, 60 min) using SYBR Gold staining. Controls included a positive control (PC, 5 × 10^4^ GE from L. braziliensis M2904 gDNA), a human negative control (HC, human PBMC gDNA), and a no-template control (NTC). (TIF) [file pntd.0013456.s006.tif]

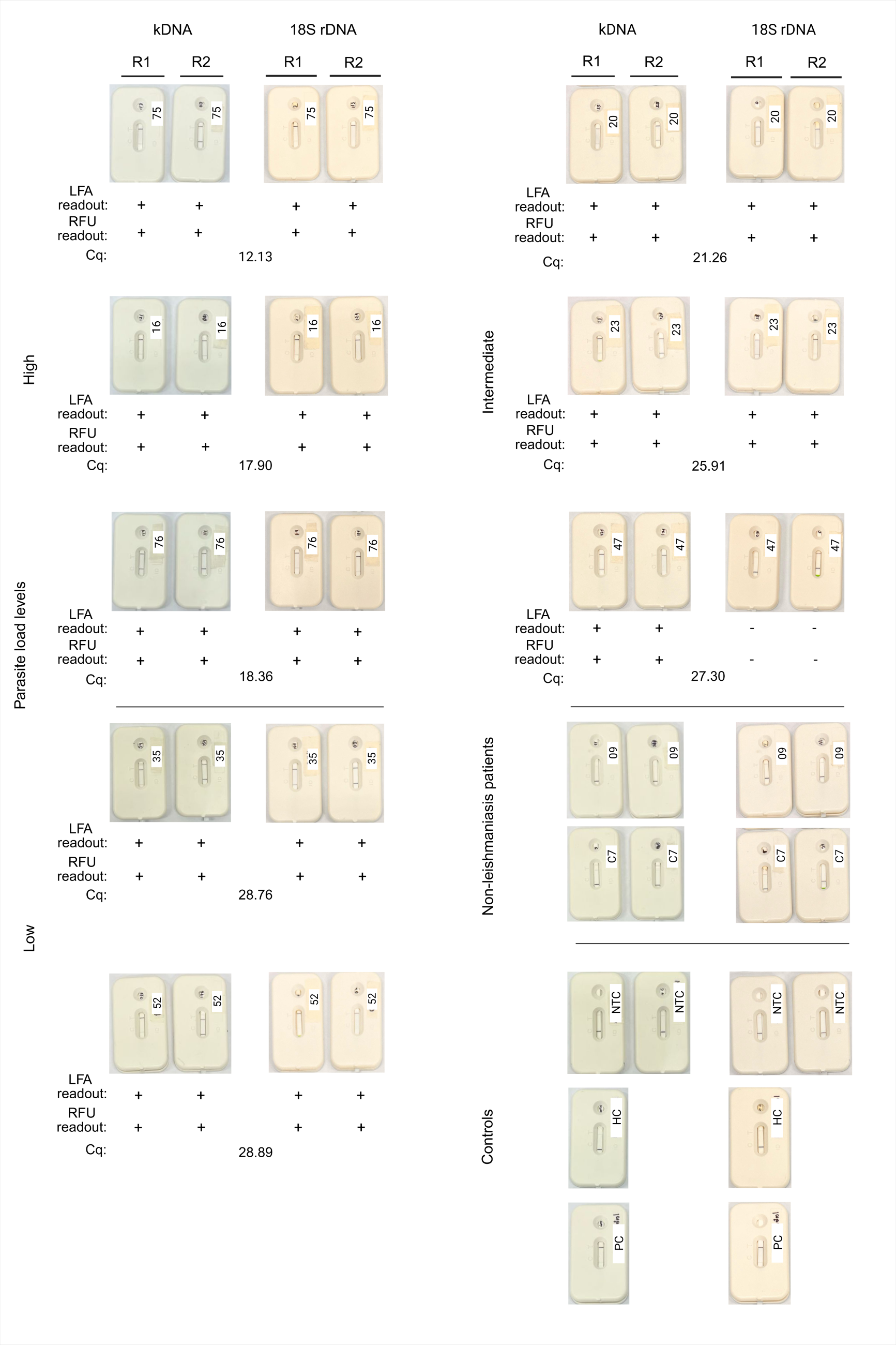

Supplement: S7 Fig — Clinical samples chosen for this pilot testing (n = 8) were categorized according to the parasite load levels and tested with kDNA and 18S LAMP-CRISPR assays. Lateral flow testing was performed on two assay repeats, R1 and R2, per sample. Visualization on the lateral flow strips was achieved through the cleavage of the biotin-FAM reporter. Following LFA analysis, the strips were placed into the cassette housing for image acquisition. C, control line; T, test line. Controls included DNA samples from two non-leishmaniasis patients (kDNA qPCR-negative; samples 09 and C7), a positive control (PC, 2 × 10^2^ GE from L. braziliensis M2904 gDNA), a human negative control (HC, 20 ng of human PBMC gDNA), and a no-template control (NTC). Photos taken by the authors. (TIF) [file pntd.0013456.s007.tif]
