# Supplementary material for: LAMP-coupled CRISPR-Cas12a assays: A promising new tool for molecular diagnosis of leishmaniasis
Source: PLoS Negl Trop Dis. 2026 Feb 13;20(2):e0013456. doi: 10.1371/journal.pntd.0013456 (PMC12923138; doi:10.1371/journal.pntd.0013456)
Supplement: S1 Table — (PDF) [file pntd.0013456.s008.pdf]

S1 Table. Primer and crRNA template sequences used in this study.

ID	Sequence 5' - 3'	Description	Application ¥	Reference
18S_crRNA	TAATACGACTCACTATAGGtaatttctactaagtgtagat GCACGGAATGAATTGAGTCA	18S rDNA dsDNA template (*)	18S crRNA synthesis	Dueñas et al., 2022
kDNA_crRNA	TAATACGACTCACTATAGGtaatttctactaagtgtagat AACGGGGTTTCTGTATGCCA	kDNA minicircle dsDNA template (*)	kDNA minicircle crRNA synthesis	Dueñas et al., 2022
18S_FIP	TGAGTCAACACTGCTGGGTGTTACGCCCAGCGAATGAATG	18S forward internal primer	18S target pre-amplification	This study
18S_BIP	GGCTTGTTCCGGCGTCTTTTGCCATGGCAGTCCACTACAC	18S backward internal primer	18S target pre-amplification	This study
18S_F3	GGCAACCATCGTCGTGAG	18S forward external primer	18S target pre-amplification	This study
18S_B3	TCGAACCCTAATCCCCCG	18S backward external primer	18S target pre-amplification	This study
18S_LF	CAGTGAAGGCATTGGTTTTACTGT	18S forward loop primer	18S target pre-amplification	This study
18S_LB	ACTGCCCTATCAGCTGGTG	18S backward loop primer	18S target pre-amplification	This study
kDNA_FIP	GGCCAAAAAACGCGAATTTTGGTGCGAAAACCGAAAAATGG	kDNA forward internal primer	kDNA target pre-amplification	This study
kDNA_BIP	CCGTGCACAATTAGGGGTTGACGACATCCTAACCCAGC	kDNA backward internal primer	kDNA target pre-amplification	This study
kDNA_F3	AATCGTACTCCCCGACAT	kDNA forward external primer	kDNA target pre-amplification	This study
kDNA_B3	GCCTTAGAGGCCAGTTTC	kDNA backward external primer	kDNA target pre-amplification	This study
kDNA_LF	GGGATTTTTGAACGGGGTTTCT	kDNA forward loop primer	kDNA target pre-amplification	This study
kDNA_LB	GTGTAATATAGTGGCCGCGCA	kDNA backward loop primer	kDNA target pre-amplification	This study
MP1L	TACTCCCCGACATGCCTCTG	kDNA forward qPCR primer	kDNA qPCR	López et al., 1993; Jara et al., 2013
MP3H	GAACGGGGTTTCTGTATGC	kDNA reverse qPCR primer	kDNA qPCR	López et al., 1993; Jara et al., 2013
PHP10-F	CATGGGAAGCAAGGGAACTAATG	ERV-3 forward qPCR primer	Human ERV-3 qPCR, for normalization of the parasite load	Yuan et al., 2001
PHP10-R	CCCAGCGAGCAATACAGAATTT	ERV-3 reverse qPCR primer	Human ERV-3 qPCR, for normalization of the parasite load	Yuan et al., 2001

(*) The dsDNA templates for crRNA generation through *in vitro* transcription were designed with a T7 promoter (underlined sequence), followed by the scaffold sequence (lowercase letter), and the specific recognition sequence (uppercase letter).

Abbreviations: rDNA, ribosomal DNA; dsDNA, double-stranded DNA; kDNA, kinetoplast DNA; ERV-3, endogenous retrovirus 3; crRNA, CRISPR RNA.
¥ Target pre-amplification refers to the LAMP amplification step prior to Cas12a-based detection.
