# Supplementary material for: LAMP-coupled CRISPR-Cas12a assays: A promising new tool for molecular diagnosis of leishmaniasis
Source: PLoS Negl Trop Dis. 2026 Feb 13;20(2):e0013456. doi: 10.1371/journal.pntd.0013456 (PMC12923138; doi:10.1371/journal.pntd.0013456)
Supplement: Attachment — Submitted filename: Response to Reviewers_MS_PNTD-D-25-01356_v2.pdf [file pntd.0013456.s014.pdf]

December 27, 2025

Dr. Alain Debrabant, Academic Editor
Dr. Sarman Singh, Section Editor
Dr. Shaden Kamhawi, co-Editor-in-Chief
Dr. Paul Brindley, co-Editor-in-Chief
PLOS Neglected Tropical Diseases

Dear editors,

Please find here enclosed a revised version of our manuscript "LAMP-coupled CRISPR-Cas12a assays for upgrading molecular detection of *Leishmania* infections" (#PNTD-D-25-01356).

The manuscript was revised according to the comments of the reviewers, and you will encounter here below a specific answer (bold) to each point addressed by the reviewers, as well as a copy of the corresponding amended text in the manuscript (italics).

We hope that we clarified all issues and look forward receiving further news from you.

Thank you for your consideration.

Sincerely,

On behalf of the authors,

Vanessa Adaui, Ph.D.
Research Group Leader in Cell Biology and Biomedicine
Laboratory of Biomolecules & School of Biology
Faculty of Health Sciences
Universidad Peruana de Ciencias Aplicadas

Reviewers' comments:
Reviewer's Responses to Questions

Key Review Criteria Required for Acceptance?

As you describe the new analyses required for acceptance, please consider the following:

Methods

-Are the objectives of the study clearly articulated with a clear testable hypothesis stated?

-Is the study design appropriate to address the stated objectives?

-Is the population clearly described and appropriate for the hypothesis being tested?

-Is the sample size sufficient to ensure adequate power to address the hypothesis being tested?

-Were correct statistical analysis used to support conclusions?

-Are there concerns about ethical or regulatory requirements being met?

Reviewer #1: -Are the objectives of the study clearly articulated with a clear testable hypothesis stated? Yes
-Is the study design appropriate to address the stated objectives? Yes
-Is the population clearly described and appropriate for the hypothesis being tested? Yes
-Is the sample size sufficient to ensure adequate power to address the hypothesis being tested? Yes
-Were correct statistical analysis used to support conclusions? yes
-Are there concerns about ethical or regulatory requirements being met? yes

Reviewer #2: The study objectives, study design, statistical analysis and conclusion are fine. The sample size for clinical evaluation of the tests is not justified.

R. We herein describe new proof-of-concept LAMP-CRISPR assays for *Leishmania* spp. detection and the initial evaluation of their diagnostic performance using a panel of 90 human clinical samples (skin lesion specimens) with real-time PCR-confirmed *Leishmania* infection status (comprising 55 *Leishmania*-positive and 35 negative).
As suggested by CLSI guidelines (CLSI, 2008), initial clinical validation studies of qualitative diagnostic assays reported in the literature typically use panels of about 100 samples (often 50 each from cases and non-cases) to provide preliminary estimates of clinical sensitivity and specificity before conducting larger studies. For example, Broughton et al. (2020, PMID: 32300245) developed a SARS-CoV-2 DETECTR assay (RT-LAMP coupled with CRISPR-Cas12a); the fluorescence version of this assay was evaluated on a total of 83 clinical samples (41 COVID-19

positive and 42 negative). Similarly, Cunningham et al. (2021, PMID: 34139428) evaluated a proof-of-concept *P. falciparum* SHERLOCK assay using 112 samples (62 PCR-positive and 50 PCR-negative).
Our sample size aligns with this practice. We added the corresponding information in the Methods section. See p. 11 (lines 228-238)
"Study samples
In this pilot laboratory study, we developed and evaluated two assays for Leishmania spp. detection using LAMP coupled with CRISPR-Cas12a. As suggested by CLSI guidelines [59], early-phase evaluation studies of qualitative diagnostic assays typically use a convenience sample of approximately 100 clinical specimens (e.g., 50 positive and 50 negative) to obtain preliminary estimates of assay performance (sensitivity and specificity). In line with this practice, a total of 90 samples were available for the current study, comprising 55 positive and 35 negative samples for Leishmania infection, as determined by a kDNA qPCR assay [60; see below]. We report herein the diagnostic sensitivity and specificity of the new assays, along with their 95% confidence intervals (CIs), which indicate the precision of these estimates."

Reference added:
59. CLSI. User protocol for evaluation of qualitative test performance. Approved guideline, 2nd ed. CLSI document EP12-A2. Wayne, PA: Clinical and Laboratory Standards Institute; 2008.

Reviewer #3: All methods used are suitable for answering the hypothesis.

Results

-Does the analysis presented match the analysis plan?

-Are the results clearly and completely presented?

-Are the figures (Tables, Images) of sufficient quality for clarity?

Reviewer #1: -Does the analysis presented match the analysis plan? yes
-Are the results clearly and completely presented? Yes
-Are the figures (Tables, Images) of sufficient quality for clarity? Yes

Reviewer #2: The analysis and presentation of the results are clear and tables and figures as well.

Reviewer #3: All results presented are in line with the analysis plan and are clearly presented.

Conclusions

-Are the conclusions supported by the data presented?

-Are the limitations of analysis clearly described?

-Do the authors discuss how these data can be helpful to advance our understanding of the topic under study?

-Is public health relevance addressed?

Reviewer #1: -Are the conclusions supported by the data presented? yes
-Are the limitations of analysis clearly described? yes
-Do the authors discuss how these data can be helpful to advance our understanding of the topic under study? yes
-Is public health relevance addressed? yes

Reviewer #2: The conclusion is supported by the obtained results. Limitation of the study is that it needs further improvement to comply with WHO ASSURED criteria. They, however, need to indicate approximate cost of the current version of the tests and how they plan to bring it down so that it can be implemented in the LMIC if eventual phase III diagnostic trial confirmed tests' diagnostic accuracy more than 95%.

R. In the Discussion section, we have acknowledged the limitations of our study as a proof-of-concept. We have expanded the discussion on considerations and challenges for the implementation of these assays in field settings. We added the requested cost estimation of our laboratory CRISPR assays and outlined potential strategies to achieve cost-effective diagnostic tests, aiming to enable implementation in LMICs. See p. 36-37 (lines 852-866)
"Another critical factor is the cost of CRISPR-based assay development and validation. The estimated per-sample cost for our laboratory LAMP-CRISPR assays was USD 13.4 with an LFA readout and USD 11.2 with a fluorescence readout, compared to USD 18.3 for kDNA qPCR (see S2 Table). The largest cost component (USD 3.2 per reaction) derives from commercial enzymes in the LAMP master mix kit, which could be reduced through scaling. One alternative is large-scale local production of recombinant enzymes for LAMP and CRISPR reactions; however, this would require additional investment in lot-to-lot analytical validation to ensure consistent reagent performance and to meet regulatory standards for in vitro diagnostic applications. The cost of the Milenia HybriDetect LFA kit (USD 2.7 per test) may be brought down through high-volume procurement. In this context, a cost-effective strategy may involve sourcing reagents and consumables in large quantities through supply chains embedded within the healthcare system. Ensuring sustained availability and affordability in low- and middle-income countries will require the integration of CRISPR-based tools and other molecular diagnostics into national health policies."

We have added a new supplementary table (S2 Table), which presents the cost estimation of our developed assays.

Reviewer #3: All conclusions are corroborated by the data presented and have an impact on public health. The authors discuss the limitations of the analysis.

Editorial and Data Presentation Modifications?

Use this section for editorial suggestions as well as relatively minor modifications of existing data that would enhance clarity. If the only modifications needed are minor and/or editorial, you may wish to recommend "Minor Revision" or "Accept".

Reviewer #1: NA

Reviewer #2: The current version of the manuscript is too long. Repeats can be avoided. Technical details can be summarized where applicable.

R. We have attempted to shorten the manuscript and have carefully revised the text to remove repetitions. The methods section remains detailed to ensure that the experiments can be readily reproduced by other researchers.

Reviewer #3: The manuscript presents robust results on the use of LAMP combined with CRISPR-Cas12a for the diagnosis of cutaneous leishmaniasis. The two assays evaluated, particularly the one using the kDNA target, showed good analytical and clinical performance. My suggestion is that the article be accepted after minor modifications.
Comments:
1- Include in the Methods section the criterion (qPCR kDNA) used to define cases and non-cases of cutaneous leishmaniasis;

R. We have included this information in the Methods section as follows. See p. 11 (lines 234-236)
***"In line with this practice, a total of 90 samples were available for the current study, comprising 55 positive and 35 negative samples for Leishmania infection, as determined by a kDNA qPCR assay [60; see below]**."*

See also p. 11 (lines 240-245)
"Quantitative real-time PCR (qPCR)
An in-house qPCR assay [60] targeting a kDNA minicircle region conserved among L. (Viannia) species [61] served as the reference test. The human ERV-3 gene was amplified in parallel using the primers reported by Yuan et al. [62]; it served as an indicator of specimen adequacy and to estimate the parasite load normalized to the number of human cell equivalents per sample [60]."

2- Do the authors have information on the diseases of the patients in the non-case group? If so, it would be helpful to include this information in the supplementary table;

R. Unfortunately, information regarding the other etiology (non-CL) for patients in the non-case group is not available. Patients were recruited in Cusco during

2019 and 2020. Due to the COVID-19 pandemic, many patients did not return for medical follow-up or additional laboratory testing.

3- Were parasitological tests (imprint, scarification, or culture) performed on the patients? If so, it would be important to compare the performance of these techniques with the results obtained with the LAMP-CRISPR-Cas12a assays;

R. No parasitological tests (imprint, dermal scraping smears, or culture) were performed. Our study analyzed stored skin lesion samples from patients with *Leishmania* infection status confirmed by a highly sensitive and specific real-time PCR (kDNA qPCR). Molecular tests are in principle more sensitive than microscopy or culture for the diagnosis of CL (e.g. Adams et al., 2014, PMID: 25111885). Nevertheless, we agree that it will be important to assess the performance of the new LAMP-CRISPR assays against parasitological tests, and ideally against a composite reference standard that combines multiple laboratory tests (because no perfect reference standard exists for leishmaniasis diagnosis). Such an evaluation would require a prospective study with a larger number of consecutively enrolled patients with clinically suspected CL to determine diagnostic accuracy under real-world conditions.

4- Although the authors suggest that the CRISPR-Cas12a system increases the specificity of the reaction, it would be interesting to demonstrate the results of the 18S LAMP and kDNA assays without the use of the CRISPR system. This comparative analysis would demonstrate the real need to use CRISPR to increase specificity;

R. This is a very relevant suggestion. We agree that it would be interesting to assess the specificity of our 18S and kDNA LAMP assays alone compared to when they are coupled with CRISPR-Cas12a. We did not formally assess this in our current study because our focus was on developing assays with a CRISPR-based detection readout. Being aware of prior literature already showing that LAMP alone is prone to non-specific amplification (e.g. Wang et al., 2015, PMID: 25853320), we did analyze LAMP products for a group of samples by endpoint visualization using agarose gel electrophoresis. We noted ladder-like bands that were ambiguous or difficult to interpret, particularly in the 18S LAMP assay (see S6 Fig). When these same amplicons were subjected to CRISPR-based detection, the CRISPR-Cas12a system provided clear discrimination between positive and negative amplification results, in line with previous studies (Gutierrez Guarnizo et al., 2025, PMID: 41085330; Sen et al., 2024, PMID: 38653014). We have stated this in the Results (see p. 25, lines 572-577) and Discussion (see p. 32-33, lines 760-766). In light of the reviewer's suggestion, we have amended the manuscript title as follows, to accurately reflect the content and contribution of our study: "*LAMP-coupled CRISPR-Cas12a assays: a promising new tool for molecular diagnosis of leishmaniasis*".

5- Include in the discussion other articles that evaluated Lamp for cutaneous leishmaniasis, without the use of the CRISPR-Cas12a system.

R. We believe that the main articles evaluating laboratory-developed LAMP assays (see references 88, 89, 91) as well as the commercially available Loopamp *Leishmania* detection kit (see references 22, 97, 100, 101) for the diagnosis of cutaneous leishmaniasis were already included in the discussion. However, after reviewing the literature, we considered relevant to include two additional studies concerning the evaluation of LAMP assay performance for New World CL (León et al., 2018, PMID: 29532767; and Soares et al., 2024, PMID: 39172895). See p. 31-32 (lines 728-749)

"Several studies have developed LAMP assays for detecting Leishmania infections in diagnosing human VL, CL, and post-kala-azar dermal leishmaniasis (PKDL), as well as in domestic animal reservoirs (dogs) and sand flies. They target multicopy genomic regions, such as kDNA minicircles [86-88], the 18S rRNA gene [89-91], the ITS1 region in the rDNA array [92], cysteine proteinase B genes (cpb) [93], and the heat shock protein 70 gene (HSP70) [94]. These assays proved to be highly sensitive for the detection of VL- and CL-causing Leishmania species (at the genus, species complex, or species level, depending on the target used). To date, few studies have evaluated the diagnostic performance of LAMP assays for New World CL. Nzelu et al. developed a colorimetric LAMP assay using malachite green that targets the Leishmania 18S rRNA gene [90]. This assay could reliably amplify Leishmania DNA from patients' skin lesion material spotted onto FTA cards [91]. In an evaluation of 122 clinical samples from CL patients in Peru, the FTA-LAMP detected 71/122 (58.2%) samples, performing comparably to nested PCR [91]. León et al. [95] assessed the same LAMP assay [90] on direct smears (n= 50) from suspected CL patients in Colombia, reporting a sensitivity of 100% (95% CI: 98.7-100%) and a specificity of 90.9% (95% CI: 69.4-100%) relative to qPCR. More recently, in Brazil, Soares et al. [94] developed a LAMP assay targeting the Leishmania HSP70 gene and evaluated its diagnostic accuracy in 100 skin biopsy samples (60 CL cases and 40 non-cases) against qPCR. The LAMP-Leish/HSP70 assay achieved a sensitivity of 86.7% (95% CI: 75.4-94.1%), a specificity of 100% (95% CI: 91.2-100%), and an overall accuracy of 92% (95% CI: 84.8-96.5%). Taken together, these studies underscore the potential of LAMP as a diagnostic and surveillance tool in endemic regions."

References added:

94. Soares ARC, Faria VCS, Avelar DM. Development and accuracy evaluation of a new loop-mediated isothermal amplification assay targeting the HSP70 gene for the diagnosis of cutaneous leishmaniasis. PLoS One. 2024 Aug 22;19(8):e0306967.

95. León CM, Muñoz M, Tabares JH, Hernandez C, Florez C, Ayala MS, et al. Analytical Performance of a Loop-Mediated Isothermal Amplification Assay for Leishmania DNA Detection in Sandflies and Direct Smears of Patients with Cutaneous Leishmaniasis. Am J Trop Med Hyg. 2018 May;98(5):1325-1331.

We also expanded the discussion on two studies that have evaluated the efficacy of the Loopamp assay for CL diagnosis in the New World. See p. 33 (lines 776-785)

"So far, two studies have evaluated the efficacy of the Loopamp assay for CL diagnosis in the New World. Adams et al. [97] conducted a prospective cohort study on 105 clinically suspected CL patients in Colombia. Using lesion swab samples, the Loopamp assay achieved a sensitivity of 95% (95% CI: 87.2-98.5%) and a specificity of 86% (95% CI: 67.3-95.9%) compared with a composite reference standard of microscopy and/or culture. In Suriname, Schallig et al. [101] assessed 93 suspected

CL cases. The assay had a sensitivity of 84.8% (95% CI: 75.0-91.9%) compared to microscopy and 91.4% (95% CI: 83.0-96.5%) compared to PCR. Specificity was moderate against microscopy (42.9%, 95% CI: 17.7-71.1%) but substantially higher when compared with PCR (91.7%, 95% CI: 61.5-99.8%).”

Summary and General Comments

Use this section to provide overall comments, discuss strengths/weaknesses of the study, novelty, significance, general execution and scholarship. You may also include additional comments for the author, including concerns about dual publication, research ethics, or publication ethics. If requesting major revision, please articulate the new experiments that are needed.

Reviewer #1: The manuscript presents the development and validation of CRISPR–Cas12a-based molecular assays coupled with LAMP amplification for the detection of Leishmania spp. The study is well designed, clearly written, and makes a significant contribution to the field of molecular diagnostics for tegumentary leishmaniasis, particularly in the context of low-resource endemic settings. The work is timely and relevant, and the results are convincingly presented. I have only minor comments and suggestions that may help strengthen the manuscript:

Contextualization of field implementation
The authors may wish to expand slightly on potential challenges or considerations for the implementation of these assays in real-world field settings (e.g., sample preparation, training needs, device portability, or cold chain requirements). This would better highlight the practical applicability of the tool.

R. Following the suggestion of the reviewer, we have expanded the discussion on considerations and challenges for the implementation of these assays in field settings. See p. 35-37 (lines 826-879)
“Currently, our proof-of-concept workflow requires controlled laboratory conditions. Future work should consider adapting these assays to meet the ASSURED/REASSURED criteria for a diagnostic test suitable for adoption across different healthcare levels, which require assays to be affordable, sensitive, specific, user-friendly, robust, rapid, as well as equipment-free (or operating with minimal equipment), deliverable, with real-time connectivity, and workable on easily collected specimens [105, 106]. A major challenge for the implementation of molecular diagnostics in resource-limited settings is sample preparation [107]. Therefore, optimizing rapid DNA extraction protocols, such as boil-spin [102] and SpeedXtract (a magnetic bead-based method) [108], combined with a streamlined CRISPR assay workflow, will be key for advancing toward a near-PoC nucleic acid-based diagnostic assay for CL and other forms of leishmaniasis. Both LAMP and CRISPR reactions are compatible with freeze-drying, which enhances reagent stability for easy transport and storage at room temperature and may reduce contamination risks [109]. This approach could enable a pre-designed kit format for portable assay setup in resource-limited settings, though its impact on assay performance must be evaluated. Incorporating these refinements alongside user-friendly devices, such as LFA strips or a blue-light transilluminator, would facilitate adoption in minimally-equipped laboratories in

endemic regions, and even in a mobile suitcase laboratory. The latter approach has been explored with an RPA assay in field studies conducted in Asia, showing promising performance for VL, PKDL, and CL diagnosis [108, 110, 111]. Another challenge for implementing these assays in field settings is ensuring continuous training and competency of personnel, particularly given the high turnover among staff. To maintain reliable performance, training programs should not only cover routine test procedures but also emphasize quality control and troubleshooting skills [112]. These training needs can be greatly reduced if the reaction setup and signal readout are integrated into a self-contained, user-friendly device [81]. Another critical factor is the cost of CRISPR-based assay development and validation. The estimated per-sample cost for our laboratory LAMP-CRISPR assays was USD 13.4 with an LFA readout and USD 11.2 with a fluorescence readout, compared to USD 18.3 for kDNA qPCR (see S2 Table). The largest cost component (USD 3.2 per reaction) derives from commercial enzymes in the LAMP master mix kit, which could be reduced through scaling. One alternative is large-scale local production of recombinant enzymes for LAMP and CRISPR reactions; however, this would require additional investment in lot-to-lot analytical validation to ensure consistent reagent performance and to meet regulatory standards for in vitro diagnostic applications. The cost of the Milenia HybriDetect LFA kit (USD 2.7 per test) may be brought down through high-volume procurement. In this context, a cost-effective strategy may involve sourcing reagents and consumables in large quantities through supply chains embedded within the healthcare system. Ensuring sustained availability and affordability in low- and middle-income countries will require the integration of CRISPR-based tools and other molecular diagnostics into national health policies.

After further optimization, streamlining, and validation, we envisage that our assays may become valuable tools for providing rapid testing results directly in affected regions, reducing delays associated with testing in centralized laboratories and supporting real-time epidemiological surveillance. Looking ahead, integrating our assays with lab-on-chip systems, such as the paper-based LAMP-CRISPR integrated diagnostic platform (PLACID) [37], could significantly enhance field applicability. PLACID uses freeze-dried reagents and paper microfluidics to perform amplification and detection without fluid transfer, with fluorescence readout via smartphone. This platform showed robust performance for bacterial and viral targets without false positives [37]. These refinements represent important considerations for future assay development. Indeed, our current two-step LAMP-CRISPR open assay format carries an inherent risk of cross-contamination during sample transfer from tube-based (LAMP) to plate-based (CRISPR) reactions, as reported in other studies [109].

Reference added:

112. Campbell S, Anderson N, Babady E, Durant TJS, Fisher A, Moore N. Centralized vs Decentralized Molecular Infectious Disease Testing. Clin Chem. 2021 Apr 29;67(5):713-719.

Clarification of assay comparison

The comparison with reference qPCR methods is clearly described. It could be helpful to add a brief statement on the Ct range or parasite loads of the discordant samples to further illustrate assay performance in low-parasite contexts.

R. This is a pertinent suggestion. The requested information was already included in the original manuscript (p. 25, lines 585-590):
"The five qPCR-positive samples not detected by the kDNA LAMP-CRISPR assay (codes 3, 5, 6, 28, 71) harbored low parasite loads (≤20 parasites per 10^6 human cells), corresponding to 10^{-2} to 10^{-3} parasite GE per reaction (Fig 6B, S1 Dataset). Similarly, the 15 qPCR-positive samples not detected by the 18S LAMP-CRISPR assay contained ≤150 parasites per 10^6 human cells, corresponding to 10^{-1} to 10^{-3} parasite GE per reaction (Fig 6D, S1 Dataset)."

Discussion on 18S assay performance
The lower sensitivity of the 18S assay compared to kDNA is briefly noted. A short discussion of potential biological or technical reasons for this difference (e.g., target copy number, sequence diversity) would be valuable for readers considering assay selection.

R. This is a good point. The requested information was already included in the original manuscript (p. 31, lines 723-727):
"*The 18S assay showed a somewhat reduced sensitivity when applied to clinical samples. This is likely related to differences in target copy number: Leishmania parasites present an estimated 10 to 170 copies of the nuclear 18S rDNA region per genome [83, 84], while they harbor about 10,000 copies of extrachromosomal kDNA minicircles per cell [85].*"
Additionally, we inspected the sequence conservation of the 18S targeted genomic region by multiple sequence alignment and found it to be highly conserved at the *Leishmania* genus level (see Results, p. 20, lines 456-459).

Minor editorial points

Ensure consistency in the use of species names (e.g., Leishmania (Viannia) vs. L. (Viannia)).

R. We have amended the text accordingly.

A few typographical issues were noted (e.g., "crossreactivity" should be "cross-reactivity"). A final language check is recommended.

R. We have checked the grammar and spelling of the English words and modified the text when required.

Overall, this is an excellent manuscript that presents innovative diagnostic tools with clear public health relevance. With minor clarifications, it will make a strong contribution to the literature on Leishmania diagnostics.

R. We thank the reviewer for the positive evaluation of our work and for the insightful suggestions, which have helped improve our manuscript.

Reviewer #2: The study is encouraging to pave the way to take molecular test in a lateral-flow test. However, the proposed DNA amplification tools are challenging in the field which limits its eventual implementation in the LMIC. The sensitivity is

R. We agree with the reviewer. We have expanded our discussion on considerations and challenges for the implementation of these assays in field settings. See p. 35-37, lines 826-879.

Reviewer #3: Comments above
